# Wutou decoction attenuates rheumatoid arthritis by modulating the Ahr/LOC101928120/SHC1 pathway

**DOI:** 10.1080/13880209.2021.1941131

**Published:** 2021-06-29

**Authors:** Dan Wu, Xi Li, Jun Liu, Can Hu, Jiefang Li

**Affiliations:** Traditional Chinese Medicine Department, The Fourth Hospital of Changsha, Changsha, Hunan, China

**Keywords:** HDAC1, histone deacetylation, transcription factor, ROS production, RNA pull-down

## Abstract

**Context:**

Wutou decoction (WTD) is a Chinese herbal formula alleviating rheumatoid arthritis (RA). SHC adaptor protein 1 (SHC1) regulates apoptosis, inflammation, and the production of reactive oxygen species (ROS). The *LOC101928120* gene is located near the *SHC1* gene. Bioinformatics analysis showed that the long non-coding RNA LOC101928120 binds to histone deacetylase HDAC1 that might regulate SHC1 expression. The *LOC101928120* gene might be targeted by the transcriptional factor Aryl hydrocarbon receptor (Ahr).

**Objective:**

This study determines the involvement of the Ahr/LOC101928120/SHC1 pathway in WTD alleviation of RA.

**Materials and methods:**

Wistar rats were injected with complete Freund’s adjuvant in the hind footpad to construe the RA model. WTD (9.8 g/kg/day) was administered intragastrically for 15 days. The CHON-001 chondrocyte cells were treated with IL-1β (10 ng/mL) alone or in combination with WTD (1 μg/mL). A RNA pull-down assay was performed to determine the interaction between LOC101928120 and HDAC1. Ahr targeting the *LOC101928120* gene was detected using luciferase reporter and chromatin immunoprecipitation assays.

**Results:**

WTD alleviated the swelling of the hind paw in rats with RA and suppressed the chondrocyte apoptosis and ROS production caused by IL-1β. WTD decreased SHC1 but increased LOC101928120 in IL-1β-treated chondrocytes. SHC1 knockdown and LOC101928120 overexpression also showed the protection. However, LOC101928120 knockdown attenuated the protective effects of WTD. WTD stimulated Ahr, which promoted LOC101928120 transcription. LOC101928120 recruited HDAC1 to the promoter region of the *SHC1* gene, thereby decreasing SHC1.

**Discussion and conclusion:**

This study revealed a new mechanism by which WTD alleviates RA by modulating the Ahr/LOC101928120/SHC1 pathway.

## Introduction

Rheumatoid arthritis (RA) is a chronic autoimmune disease associated with progressive articular damage, functional loss, and joint pain. Inflammatory cytokines, proteases, and reactive oxidative stress have been proposed to launch a cascade of events leading to clinical impairment and chronic destructive arthritis (Smolen et al. 2016; Aletaha and Smolen 2018; Littlejohn and Monrad 2018). RA affects 0.5–1% of the global population. However, no drug cures RA completely and many treatment drugs (e.g., non-steroidal anti-inflammatory drugs, glucocorticoids, and immunosuppressants) have various side effects that include gastrointestinal and liver disorders (Smolen et al. 2016; Aletaha and Smolen 2018; Littlejohn and Monrad 2018).

Wutou decoction (WTD) is a classic Chinese herbal formula that has a favourable therapeutic response in treating RA. WTD consists of *Aconiti Radix Cocta*, *Ephedrae Herba*, *Paeoniae Radix Alba*, *Astragali Radix*, and *Glycyrrhiza Radix Preparata*. Various alkaloids, triterpene saponins, and some monoterpene glycosides, flavones, and flavones glycosides have been identified in WTD (Dai et al. 2014; Cheng et al. 2020). These components may be involved in the therapeutic effect in RA. WTD decreases inflammatory factors, including interleukin-1 (IL-1) and tumour necrosis factor-alpha (TNF-α), modulates the proportions of CD4^+^/CD8^+^ in peripheral blood, and alleviates the pain of joints in patients with RA (He et al. 2018; Zhu et al. 2018).

However, the molecular basis underlying the protection of WTD against RA has not been fully elucidated. Liu et al. (2016) reported that WTD induced epigenetic modifications, such as histone modifications, which are probably associated with the anti-inflammatory effect of WTD in RA. Zhang et al. (2015) proposed a clever method to determine the potential mechanism underlying WTD protection. First, putative targets of the composite compounds in WTD were predicted by the drug CIPHER-CS. This was followed by an intersection analysis of the putative targets of WTD and known RA-related targets. The genes at the intersection were supposed to mediate the effect of WTD against RA. We were interested in the *SHC adaptor protein 1* (SHC1) gene during the intersection because SHC1 protein is implicated in the regulation of apoptosis, inflammation, and production of reactive oxygen species (ROS) (Xian et al. 2017; Wang et al. 2019). However, SHC1 has never been implicated in the effect of WTD against RA.

LncRNAs are RNAs with a length exceeding 200 nucleotides with little or no protein-coding capacity. This group of RNAs modulates gene expression through multiple mechanisms, such as epigenetics, small RNA sponging, and other transcriptional and translational regulation. The *LOC101928120* gene is located near the *SHC1* gene. Bioinformatics analysis showed that the long non-coding RNA LOC101928120 binds to histone deacetylase HDAC1 that might regulate SHC1 expression; the *LOC101928120* gene might be targeted by the transcriptional factor Aryl hydrocarbon receptor (Ahr). Transcription factors, such as Ahr and oestrogen receptor 2 (ESR2), have been reported to be affected by many herbal extracts (Jeuken et al. 2003; Jarry et al. 2005; Cvoro et al. 2007; Wang et al. 2013). Based on the analysis, we wondered whether WTD can regulate SHC1 through LOC101928120. The present study demonstrates that WTD suppressed SHC1 expression by inducing a long non-coding RNA (lncRNA), LOC101928120. The suppression of SHC1 by WTD attenuated the many detrimental effects induced by IL-1β, a key inducer of RA. The findings reveal a new mechanism by which WTD alleviates RA.

## Materials and methods

### Animal study

All procedures were performed by the ethical standards of the Fourth Hospital of Changsha and the Xiangya Medical College, Central South University. Eighteen male Wistar rats (weighing 205–240 g, specific pathogen-free grade) were obtained from the Experimental Animal Centre of Xiangya Medical College, Central South University (Changsha, China). These rats were randomly divided into three groups (*n* = 6 per group): healthy control group, RA model group (RA), and RA model treated with WTD group (RA + WTD). All rats were acclimated for 7 days before the experiments. Rats in the RA and RA + WTD groups were injected in the right hind footpad with 0.1 mL complete Freund’s adjuvant (CFA, Amyjet Scientific Inc., Wuhan, China) containing 10 mg/mL dead *Mycobacterium tuberculosis*. Rats in the RA + WTD group were additionally administered WTD extract intragastrically at a dose of 9.8 g/kg/day of the crude drug (equal to 10 mL/kg/day) for 15 days. WTD extract was provided by the Department of Traditional Chinese Medicine, the Fourth Hospital of Changsha (Changsha, China). For the healthy control group, rats were injected with 0.1 mL saline. All animals were kept under specific pathogen-free conditions during the experiments at a constant temperature of 24 ± 1 °C in a room with a 12 h light/dark cycle and *ad libitum* access to water and food. After 15 days, all the rats were intraperitoneally injected with pentobarbital sodium (50 mg/kg) before they were sacrificed. The right hind joints were excised for histological examination, PCR, and western blot assays.

### Histologic examination

Joint tissues were fixed in 10% neutral buffered formalin and decalcified for 2 weeks. Joints were processed for paraffin embedding according to the standard protocol. Sections were subsequently cut, deparaffinized, dehydrated, and stained with Safranin O/Fast Green (Sigma–Aldrich, St. Louis, MO, USA) for general evaluation of the damage to the articular cartilage.

### Bioinformatics analysis

*SHC1* gene information was obtained from the Genome Browser Gateway (http://www.genome.ucsc.edu/cgi-bin/hgGateway?redirect=manual&source=www.genome.ucsc.edu). The *SHC1* gene is located in the antisense strand of DNA and is very close to the *LOC101928120* gene. As LOC101928120 is not translated to protein, it is a lncRNA. The Genome Browser Gateway also provides information on epigenetic modifications in the promoter region of the *SHC1* gene. Transcription factors targeting the *LOC101928120* gene were predicted using Jasper 2020 (http://jaspar.genereg.net/). The interaction between LOC101928120 and HDAC1 was analyzed using online tools from catRAPID (http://s.tartaglialab.com/page/catrapid_group).

### Gene expression analysis

Polymerase chain reaction (PCR) was performed to evaluate gene expression. Total RNA was isolated from chondrocytes using TRIzol reagent (Invitrogen, Carlsbad, CA, USA) following the standard protocol. To determine the percentages of LOC101928120 in the cytoplasm and nucleus, RNA was extracted from these subcellular fractions using the Cytoplasmic and Nuclear RNA Purification Kit (Norgen Biotek, Beijing, China). The RNA was then purified through phenol-chloroform extraction and used for the synthesis of the first-strand cDNA using RevertAid H Minus First Strand cDNA Synthesis Kit (Thermo Fisher Scientific, Waltham, MD). Quantitative real-time PCR was conducted using SYBR Green PCR Master Mix (ABI 4309155; Thermo Fisher Scientific) in an ABI7900 PCR machine (Thermo Fisher Scientific). The primer sequences are shown in Table 1.

**Table 1.** Primer information.

Gene names	Direction	Sequences (5′–3′)
SHC1	Forward	TACTTGGTTCGGTACATGGGT
SHC1	Reverse	CTGAGTCCGGGTGTTGAAGTC
LOC101928120	Forward	AAGTGCGACGACAGGGAAGC
LOC101928120	Reverse	AGAAGTCAGCCCGAATGCCT
β-actin	Forward	ACCCTGAAGTACCCCATCGAG
β-actin	Reverse	AGCACAGCCTGGATAGCAAC
U1	Forward	GGGAGATACCATGATCACGAAGGT
U1	Reverse	CCACAAATTATGCAGTCGAGTTTCCC

### Western blot

Total protein was extracted using RIPA buffer (1% NP-40, 0.1% SDS, 50 mM DTT) supplemented with freshly added Aprotinin (2 µg/mL), Leupeptin (2 µg/mL), and PMSF (1 mM) on ice. Cytoplasmic and nuclear fractions of the chondrocytes were prepared according to the instructions of the Nuclear/Cytoplasmic Isolation kit (Thermo Fisher Scientific, Waltham, MA, USA). Proteins were separated by SDS-PAGE and transferred to a nitrocellulose membrane with a 0.45 µm pore size. The membranes were blocked in 5% non-fat milk and then incubated with primary antibodies against SHC1 (1:500; Abcam, Cambridge, UK), phosphorylated protein kinase B (p-AKT; 1:1000; Santa Cruz Biotechnology, Dallas, TX, USA), Raf1 (1:500; Santa Cruz Biotechnology), phosphorylated extracellular signal-regulated kinase (p-ERK1/2; 1:1000; Santa Cruz Biotechnology), Histone deacetylase 1 (HDAC1; 1:500; Abcam), Ahr (1:500; Abcam), histone 3 (1:1000; Abcam), and β-actin (1:1000; Ptgcn, Shanghai, China) at 4 °C overnight. After thorough washing with TBST (10 mM Tris, pH 8.0, 150 mM NaCl, 0.5% Tween 20), the membranes were further incubated with a 1:5000 dilution of horseradish peroxidase-conjugated secondary antibodies for 1 h at room temperature. Blots were repeatedly washed and developed with ECL substrates (Thermo Fisher Scientific).

### Cell culture and treatments

The CHON-001 chondrocyte cell line was purchased from the Chinese Academy of Sciences Cell Bank (Shanghai, China). Cells were cultured in Dulbecco’s modified Eagle’s medium (DMEM)/F12 containing 10% foetal bovine serum (FBS) in a humidified atmosphere of 5% CO_2_ at 37 °C. CHON-001 cells were stimulated with 10 ng/mL IL-1β (Sigma–Aldrich, St. Louis, MO, USA) for 24 h to establish a RA model *in vitro*.

Mao et al. (2020) previously used 0.4 μg/mL WTD to determine its protective effect in human fibroblast-like synoviocytes. According to our pilot study, we found that 1 μg/mL is a suitable dose for WTD protection in the CHON-001 chondrocytes. WTD (1 μg/mL) or the Ras inhibitor BI-3406 (1 μM) were added to CHON-001 immediately after IL-1β treatment. Small interfering RNA (siRNA) oligonucleotides targeting SHC1 and LOC101928120 were synthesized by GeneChem (Shanghai, China). *In vitro* transfection of siRNAs was conducted using Lipofectamine™ 2000 (Invitrogen; Thermo Fisher Scientific). *LOC101928120* and *Ahr* were cloned into the enhanced green fluorescent protein plasmid-C1 vector (GenePharma, Shanghai, China) to construct overexpression vectors of LOC101928120 and Ahr. Transfection of CHON-001 cells with the vectors was achieved using Lipofectamine 2000.

After the above treatments for 24 h, cells were subjected to cell viability, apoptosis, and ROS measurements.

### Cell viability evaluation

CHON-001 cells were seeded into 96-well plates at a density of 1 × 10^4^ cells per well. After the above treatments, 3‐[4,5‐dimethylthiazol‐2‐yl]‐2,5 diphenyl tetrazolium bromide (MTT; KeyGEN Biotech, Nanjing, China) was added to each well and the plates were incubated for 4 h in a 37 °C incubator. The medium was carefully removed from each well and the purple formazan was solubilized using dimethylsulphoxide (DMSO). The optical density at 590 nm (OD_590_) was determined using a Thermo Multiskan EX plate reader (Thermo Fisher Scientific).

### Cell apoptosis

Apoptosis and cell death in chondrocytes were examined by flow cytometry using Annexin V-fluorescein isothiocyanate (FITC) and propidium iodide (PI) staining. Chondrocytes were trypsinized and collected by centrifugation. After washing, the cells were double-stained with Annexin V-FITC and PI according to the manufacturer’s instructions (Solarbio, Beijing, China). The apoptosis rate was determined using a dual laser flow cytometer (Becton Dickinson, San Jose, CA, USA) with ModFitLT software (Verity Software House, Topsham, ME, USA).

### Measurement of intracellular ROS levels

Intracellular ROS levels were evaluated using 2,7-dichlorofluorescein diacetate (DCFH-DA) (Beyotime Biotechnology, Shanghai, China). DCFH-DA forms the fluorescent compound dichlorofluorescein in the presence of ROS. DCFH-DA (10 μM) was added to the cells. After incubation for 20 min at 37 °C, the cells were washed, trypsinized, and resuspended in PBS supplemented with 5% FBS. Flow cytometry analysis was performed using a Fortessa device (BD Biosciences, Santa Clara, CA, USA). A minimum of 10,000 cells was analyzed.

### RNA pull-down assay

Full-length LOC101928120 was synthesized and labelled with biotin using Biotin RNA Labelling Mix (Roche, Basel, Switzerland) and the Riboprobe Systems with T7 RNA polymerase (Promega, Madison, WI, USA). For the RNA pull-down assay, 3 µg of the purified biotin-labelled RNA probes were incubated with chondrocyte lysates for 4 h at room temperature and subsequently with streptavidin magnetic beads (Thermo Fisher Scientific) overnight at 4 °C. The proteins pulled down by LOC101928120 were further analyzed by western blot to detect the level of HDAC1 protein.

To determine the HDAC1 binding sites in LOC101928120, full-length sense and antisense LOC101928120, and specific regions of the lncRNA were synthesized and labelled with biotin using the Biotin RNA Labelling Mix. Cell lysates were incubated with the RNA probes and the standard pull-down procedure was performed. The bound proteins in the pull-down product were analyzed by western blot using HDAC1 antibody.

### Luciferase reporter assay

Wild-type *LOC101928120* gene promoter fragment was cloned into the pGL4 vector (Addgene, Inc., Cambridge, MA, USA) upstream of the firefly luciferase coding region within restriction sites XhoI and NotI (TaKaRa Bio, Inc., Otsu, Japan). Corresponding mutations were introduced into the Ahr binding site by site-directed mutagenesis using a fast mutation kit (New England BioLabs, Inc., Ipswich, MA, USA). The reporter vectors were co-transfected with the Ahr overexpression vector using Lipofectamine 2000 (Invitrogen). Twenty-four hours following transfection, the cells were lysed using Glo Lysis Buffer (E266A, Promega). The luciferase activity of each extract was assayed using the Bright-Glo Luciferase Assay System (E2620, Promega).

### Chromatin immunoprecipitation assay (ChIP)

ChIP assay was conducted to determine the acetylation status of histone 3 in the promoter region of the *SHC1* gene and the enrichment of Ahr in the promoter region of the *LOC101928120* gene. A Magna ChIP Kit (Millipore, Bedford, MA, USA) was used to extract nuclear DNA-protein complex in chondrocytes. The nuclear DNA was sonicated to produce 200–500 bp fragments. The chromatin extract was incubated with antibodies against HDAC1 (Abcam), acetylated histone 3 (Abcam), Ahr (Abcam), or IgG (Millipore). After immunoprecipitation, the DNA-protein-antibody complex was separated and the protein was removed. The purified DNA was then analyzed using qRT-PCR. The IgG and input groups were used as the negative and positive control groups, respectively.

### Statistical analyses

Data are expressed as the mean ± standard deviation of three independent experiments. A two-tailed Student’s *t*-test was performed to detect differences between two groups. The one-way ANOVA with Tukey’s *post-hoc* test was conducted to compare two-group differences among the multiple groups using GraphPad Prism 6 software (GraphPad, La Jolla, CA, USA). *p* < 0.05, *p* < 0.01, or *p* < 0.001 was considered significant.

## Results

### Attenuation of RA by WTD in the rat model

**Figure 1.**
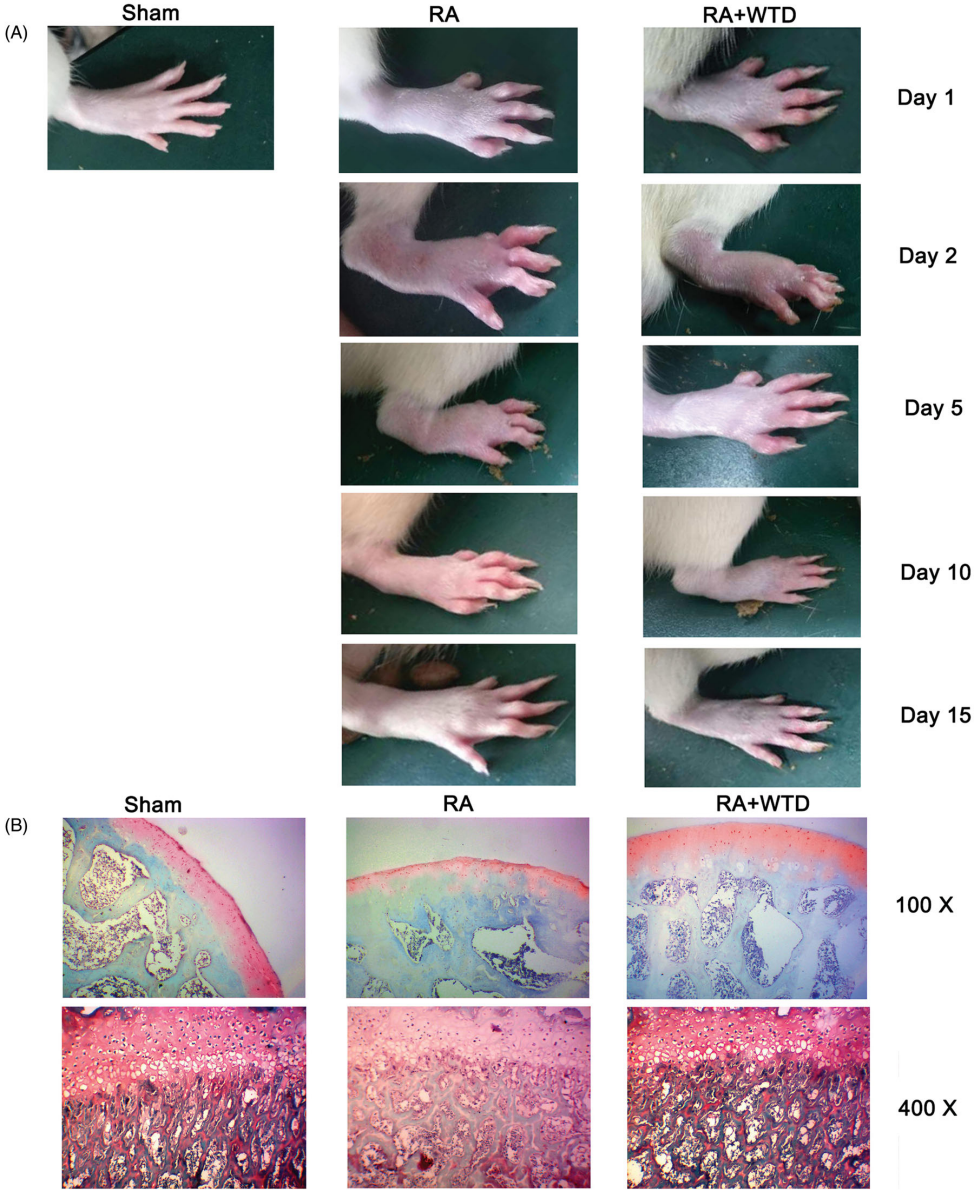
WTD attenuates RA in a rat model. Rats in the RA and RA + WTD groups were injected in the right hind footpad with 0.1 mL complete Freund’s adjuvant containing 10 mg/mL dead *Mycobacterium tuberculosis*. Rats in the RA + WTD group were additionally intragastrically administered WTD crude extract at a dose of 9.8 g/kg/day for 15 days. (A) The swelling of the hind paw was evaluated on days 1, 2, 5, 10, and 15. (B) The thickness of the articular cartilage layer in each group was evaluated by Safranin O/Fast Green staining.

Treatment with CFA induced noticeable swelling of the hind paw, especially on the second day after the CFA injection. The swelling gradually subsided within 2 weeks (Figure 1(A)). Treatment with WTD accelerated the relief of the swelling of the hind paw, which agreed with previous studies. The swelling of the hind paw in the RA + WTD group was completely relieved until day 15. However, on day 15, rats in the RA group still showed noticeable swelling of the hind paw. Therefore, we analyzed the hind joint in all groups on day 15. The effect of WTD on the cartilage in this rat model of RA was assessed using Safranin O/Fast Green staining. The thickness of the articular cartilage layer was reduced in the RA rats compared to the control rats, suggesting the loss of chondrocytes in RA (Figure 1(B)). In addition, the staining of cartilage was much weaker in the RA rats compared to the control rats. Treatment with WTD partially prevented the loss of chondrocytes in RA rats.

### WTD suppressed the elevation of SHC1 in chondrocytes induced by IL-1β

**Figure 2.**
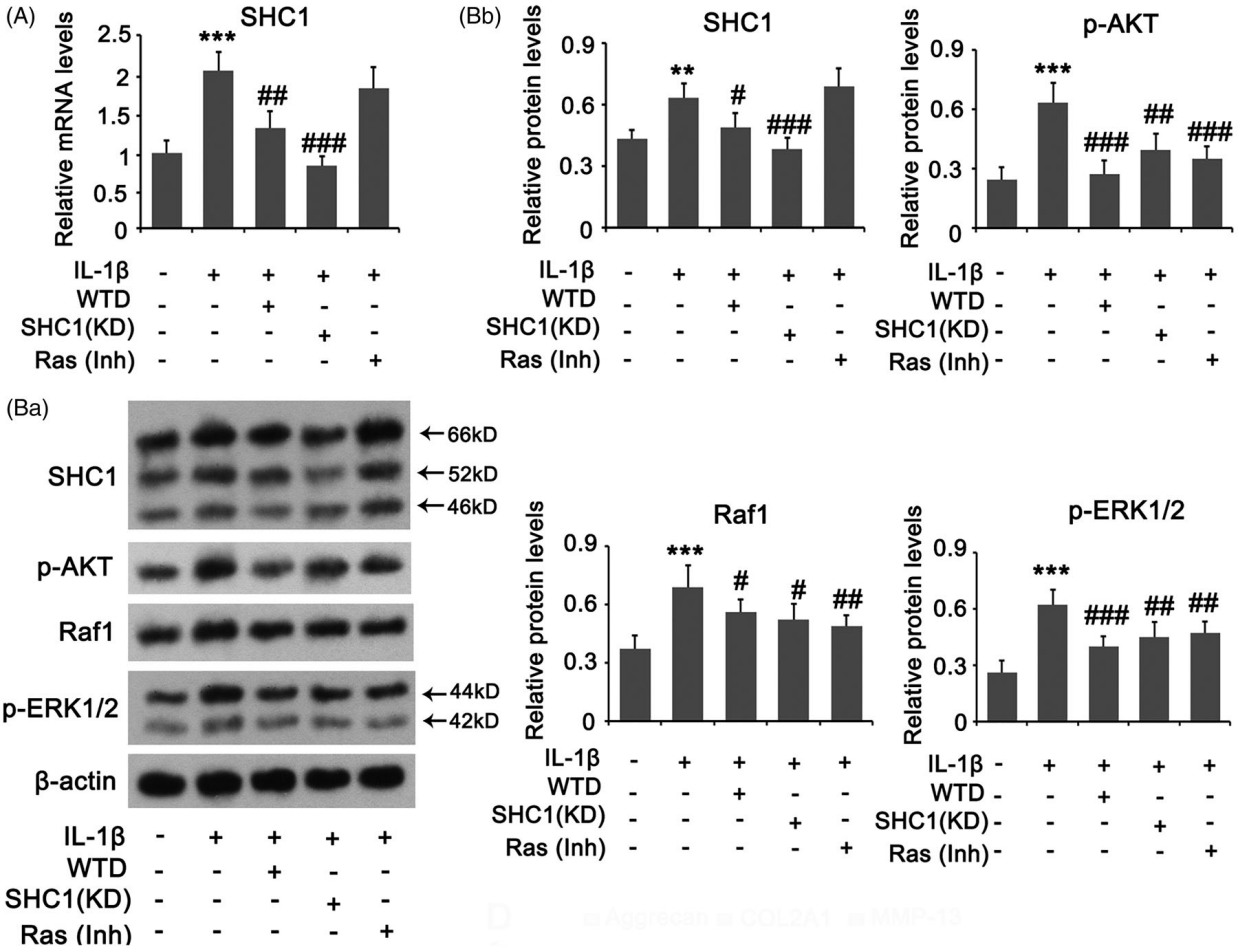
SHC1 signal in chondrocytes is regulated by IL-1β and WTD. CHON-001 cells were stimulated with 10 ng/mL IL-1β to establish the RA model. WTD (1 μg/mL) and Ras inhibitor BI-3406 (1 μM) were added to CHON-001 samples to determine their effect against IL-1β. SHC1 was knocked down in chondrocytes before exposure to IL-1β. (A) PCR and (B) western blot assays were conducted to assess the expression of the indicated genes and proteins, respectively. **p < 0.01 and ***p < 0.001 vs. Control group; ^#^*p* < 0.05, ^##^*p* < 0.01, and ^###^*p* < 0.001 vs. IL-1β group.

We constructed an *in vitro* RA model by stimulating chondrocytes with IL-1β. IL-1β induced the increase of SHC1 at both the mRNA and protein levels in chondrocytes (*p* < 0.001 and *p* < 0.01, respectively; Figures 2(A,B)). However, WTD suppressed the increase in SHC1 induced by IL-1β (*p* < 0.01 or *p* < 0.05 vs. the IL-1β group). The Ras signal is activated by the isoforms p46Shc and p52Shc of SHC1. AKT, Raf1, and ERK1/2 are downstream effectors of Ras signalling. IL-1β induced an increase in p-AKT, Raf1, and p-ERK1/2 protein levels (all *p* < 0.001, Figure 2(B)). The increases were attenuated by treatment with WTD (*p* < 0.05 or *p* < 0.001 vs. the IL-1β group). To determine the role of the SHC1/Ras pathway in IL-1β-induced RA, we knocked down SHC1 and suppressed Ras signalling by the inhibitor. SHC1 knockdown and Ras suppression also suppressed IL-1β-induced increase in p-AKT, Raf1, and p-ERK1/2 protein levels (*p* < 0.05, *p* < 0.01, *p* < 0.001 vs. the IL-1β group).

### Association of SHC1 with WTD protection against chondrocyte damage by IL-1β

**Figure 3.**
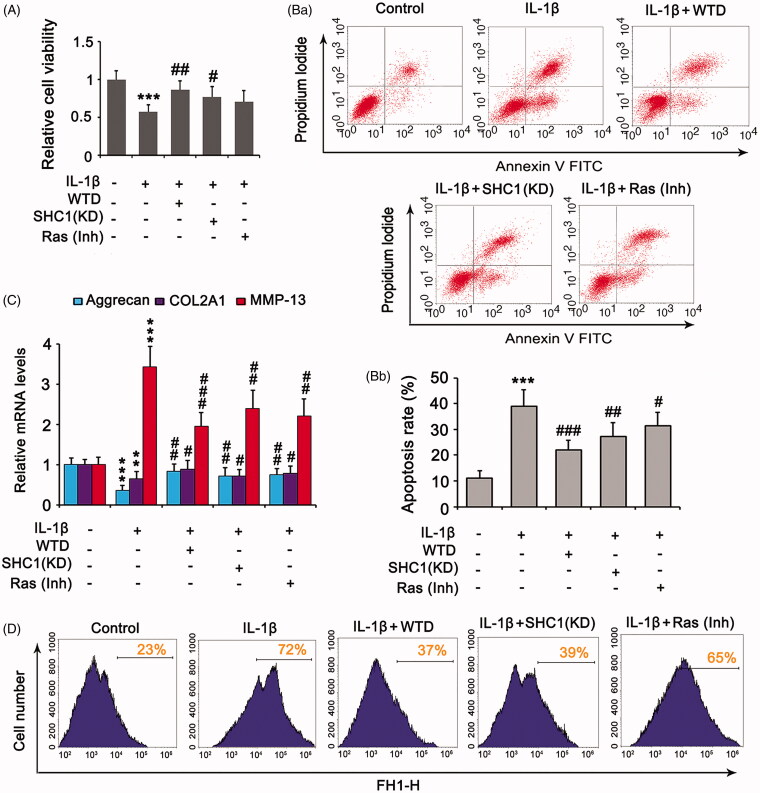
SHC1 is implicated in the protective effect of WTD against IL-1β-detrimental effects. CHON-001 cells were stimulated with 10 ng/mL IL-1β to establish the RA model. WTD (1 μg/mL) and Ras inhibitor BI-3406 (1 μM) were added to CHON-001 to determine their effect against IL-1β. SHC1 was knocked down in chondrocytes before exposure to IL-1β. (A) Cell viability was evaluated by the MTT assay. (B) Apoptosis rate was evaluated by flow cytometry. (C) Aggrecan, COL2A1, and MMP-13 expressions in chondrocytes were determined by PCR. (D) Intracellular ROS levels were evaluated by a DCFH-DA probe using flow cytometry. ***p < 0.001 vs. control group; ^#^*p* < 0.05, ^##^*p* < 0.01, and ^###^*p* < 0.001 vs. IL-1β group.

IL-1β suppressed the viability of chondrocytes (*p* < 0.001, Figure 3(A)), elevated the percentage of apoptotic cells (*p* < 0.001, Figure 3(B)), lowered the expressions of aggrecan (*p* < 0.001, Figure 3(C)) and COL2A1 (*p* < 0.01), increased the expression of matrix metalloproteinase-13 (MMP-13) expression (*p* < 0.001), and increased ROS production (Figure 3(D)). Treatment with WTD reversed these effects of IL-1β (*p* < 0.05, *p* < 0.01, or *p* < 0.001 vs. the IL-1β group). Knockdown of SHC1 attenuated IL-1β-suppressed cell viability (*p* < 0.05 vs. IL-1β group, Figure 3(A)), while suppression of Ras signalling had a weak effect in this respect. IL-1β-induced apoptosis was attenuated by the knockdown of SHC1 (*p* < 0.01 vs. IL-1β group, Figure 3(B)) and suppression of Ras signalling (*p* < 0.05 vs. the IL-1β group). The regulatory effects of IL-1β on aggrecan, COL2A1, and MMP-13 were also reduced by both SHC1 knockdown and Ras inhibition (Figure 3(C)). Depletion of SHC1 inhibited the production of ROS induced by IL-1β, whereas suppression of Ras only moderately decreased IL-1β-induced ROS (Figure 3(D)).

### WTD suppresses SHC1 expression in chondrocytes by inducing LOC101928120

**Figure 4.**
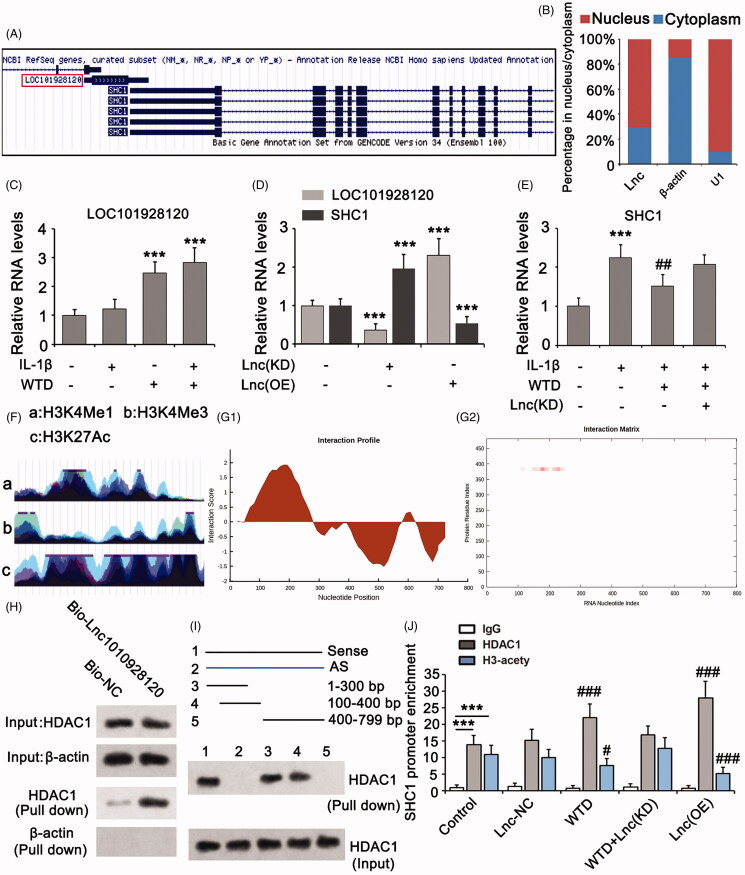
WTD suppresses SHC1 expression in chondrocytes by inducing LOC101928120. (A) Genome Browser Gateway analysis showed that the *SHC1* gene is located in the antisense strand of DNA near the *LOC101928120* gene in the sense strand. (B) PCR was performed to detect the percentage of LOC101928120 in the nucleus and cytoplasm. U1: U1 snRNA. (C) PCR was performed to detect the LOC101928120 expression in chondrocytes after treatments with IL-1β and/or WTD. ***p < 0.001 vs. control group. (D) PCR was performed to detect LOC101928120 and SHC1 expressions in chondrocytes after LOC101928120 knockdown and overexpression. ***p < 0.001 vs. control group. € PCR was performed to detect SHC1 expression in chondrocytes after treatments with IL-1β and WTD and knockdown of LOC101928120. ***p < 0.001 vs. control group; ^##^*p* < 0.01 vs. IL-1β group. (F) Genome Browser Gateway analysis showed that the promoter of the *SHC1* gene is vulnerable to histone methylation and acetylation. (G) The interaction of LOC101928120 with HDAC1 was analyzed using the catRAPID web. (H) RNA pull-down assay was conducted to confirm the interaction between LOC101928120 and HDAC1. (I) In another RNA pull-down assay, several regions of Bio-LOC101928120 and the antisense of LOC101928120 were used to pull down HDAC1. Only the sequences LOC101928120 from 1 to 300 bp and 100 to 400 bp pulled down HDAC1. (J) ChIP assay was performed to determine the interaction of HDAC1 and acetylated Histone 3 with the promoter of the *SHC1* gene. ***p < 0.001 vs. IgG group; ^#^*p* < 0.05, and ^###^*p* < 0.001 vs. control group.

Analysis of the Genome Browser Gateway showed that the *SHC1* gene is located in the antisense strand of DNA, which is near the *LOC101928120* gene in the sense strand (Figure 4(A)). As a lncRNA, LOC101928120 accumulated more in the nuclei of cells as determined by PCR (Figure 4(B)). U1 snRNA is a kind of low molecular mass RNA rich in uridine acid. U1 snRNA primarily exists in the nuclei of eukaryotic cells, thus it was used as a control in the PCR assay. IL-1β had no effect on LOC101928120 expression in chondrocytes. However, WTD remarkably increased LOC101928120 expression regardless of treatment with IL-1β or not (*p* < 0.001, Figure 4(C)). In chondrocytes, knockdown of LOC101928120 increased SHC1 expression (*p* < 0.001, Figure 4(D)), while overexpression of LOC101928120 decreased SHC1 expression (*p* < 0.001 vs. the IL-1β group). Knockdown of LOC101928120 abolished the inhibitory effect of WTD on SHC1 expression induced by IL-1β (Figure 4(E)). These results suggested that WTD suppresses SHC1 expression in chondrocytes by inducing LOC101928120. Further analysis of the Genome Browser Gateway revealed the vulnerability of the promoter of the *SHC1* gene to histone methylation and acetylation (Figure 4(F)). Therefore, we analyzed the interaction of LOC101928120 with a series of enzymes that are responsible for histone methylation and acetylation using catRAPID. LOC101928120 displayed a high affinity for the HDAC1, a histone 3 deacetylase. The detailed binding site of the HDAC1 protein in LOC101928120 was predicted to range from 100 to 400 bp (Figure 4(G)). To identify the prediction, we performed an RNA pull-down assay. Bio-LOC101928120 pulled down HDAC1, whereas Bio-NC failed to do so (Figure 4(H)). We further used several regions of Bio-LOC101928120 and the antisense of LOC101928120 to pull down HDAC1. Only the LOC101928120 sequences from 1 to 300 bp and 100 to 400 bp pulled down HDAC1 (Figure 4(I)), suggesting that the binding site of the HDAC1 protein in LOC101928120 was from 100 to 400 bp. We conducted a ChIP assay to determine whether WTD influences the acetylation status of histone 3 in the promoter region of the *SHC1* gene by inducing LOC101928120. The *SHC1* gene promoter sequence was detected in protein-DNA complexes that were isolated by both anti-HDAC1 and anti-acetylated histone 3 antibodies (Figure 4(J)). Treatment with WTD increased the enrichment of the *SHC1* gene promoter in the protein-DNA complex isolated by anti-HDAC1 antibody (*p* < 0.001) but decreased the enrichment in the protein-DNA complex isolated by anti-acetylated histone 3 antibody (*p* < 0.05), which suggested that WTD induces the interaction between HDAC1 and *SHC1* gene promoter, resulting in decreased acetylation in histone 3 in this DNA area. However, this effect of WTD was abolished after the depletion of LOC101928120. Our results collectively suggested that WTD induces the upregulation of LOC101928120, which recruits HDAC1 to the *SHC1* gene promoter and results in the deacetylation of histone 3.

### WTD increases LOC101928120 expression in chondrocytes by activating Ahr

**Figure 5.**
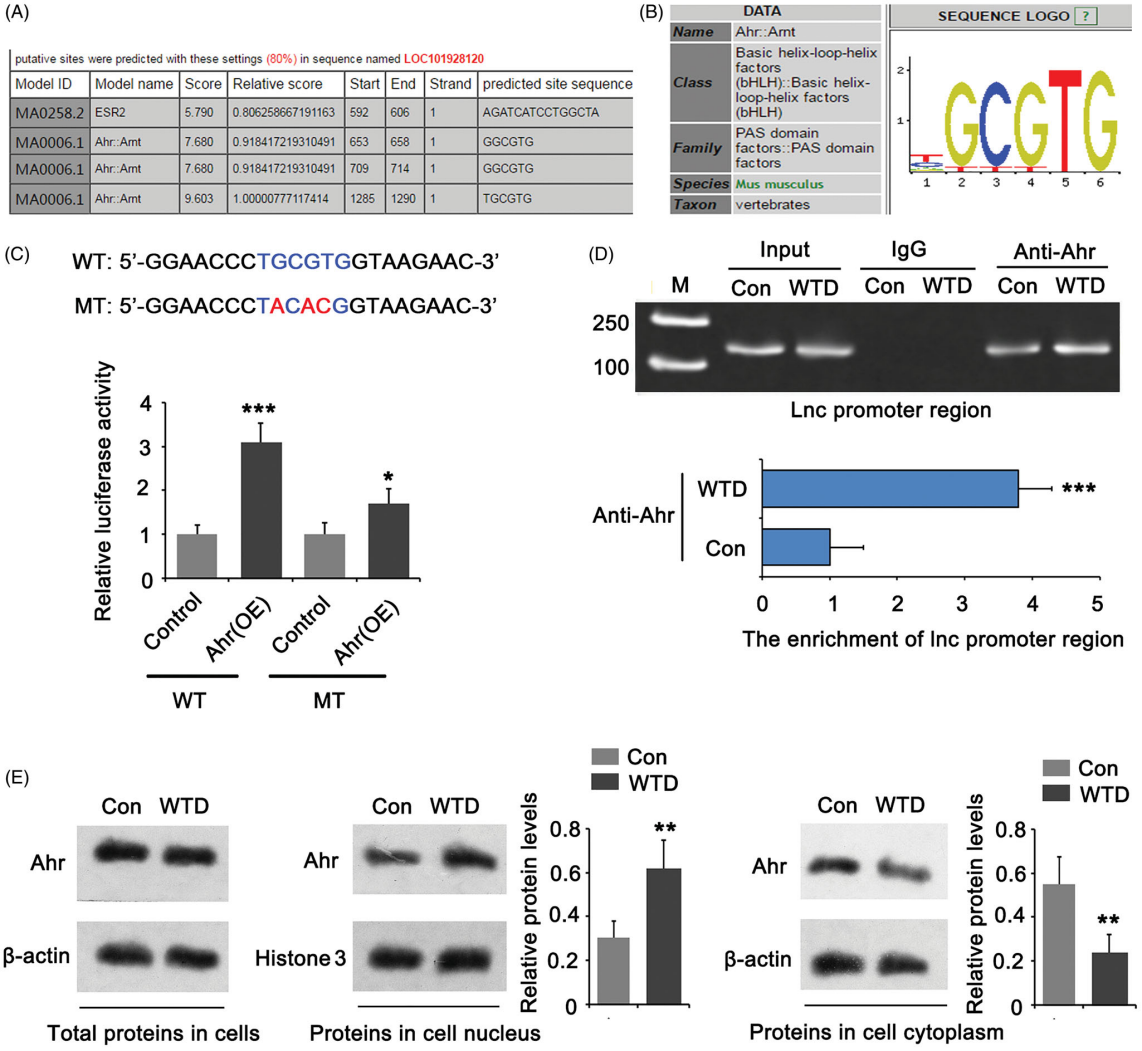
WTD up-regulates LOC101928120 by stimulating Ahr. (A) Bioinformatics analysis using Jasper 2020 showing that the *LOC101928120* gene is likely a target of Ahr. (B) The gene sequence is specifically targeted by Ahr. (C) A luciferase reporter assay was performed to confirm the interaction of Ahr with the promoter region of *LOC101928120* gene from −1285 to −1290 bp, as this region showed the highest score in the bioinformatics analysis. (D) ChIP assay was conducted to determine whether the interaction between Ahr and *LOC101928120* gene promoter is influenced by WTD. (E) Western blot assay was performed to determine the protein level of Ahr in chondrocytes and the cytoplasm and cell nucleus after WTD treatment. **p < 0.01 vs. control group; *p < 0.05, ***p < 0.001 vs. control group.

Transcription factors, such as Ahr and ESR2, have been reported to be affected by many herbal extracts. Bioinformatics analysis showed that LOC101928120 is more likely regulated by Ahr (Figures 5(A,B)). We performed a luciferase reporter assay to confirm the interaction of Ahr with the promoter region of *LOC101928120* gene from −1285 to −1290 bp, as this region showed the highest score in the bioinformatics analysis. Overexpression of Ahr increased the luciferase activity of the WT reporter (*p* < 0.001, Figure 5(C)). Mutation at the promoter region (from −1285 to −1290 bp) notably decreased the effect of Ahr overexpression on luciferase activity. The ChIP assay was conducted to determine whether the interaction between Ahr and *the LOC101928120* gene promoter is influenced by WTD. The results showed that treatment with WTD promoted the binding of Ahr to the *LOC101928120* gene promoter (*p* < 0.001, Figure 5(D)). Upon activation, Ahr was translocated from the cytoplasm to the cell nucleus. Treatment with WTD did not increase the total protein level of Ahr in chondrocytes, compared to the control (Figure 5(E)). The treatment did increase the enrichment of Ahr in the cell nucleus (*p* < 0.01) with the reduction of Ahr in the cytoplasm (*p* < 0.01).

### The protective effect of WTD against IL-1β is associated with LOC101928120

**Figure 6.**
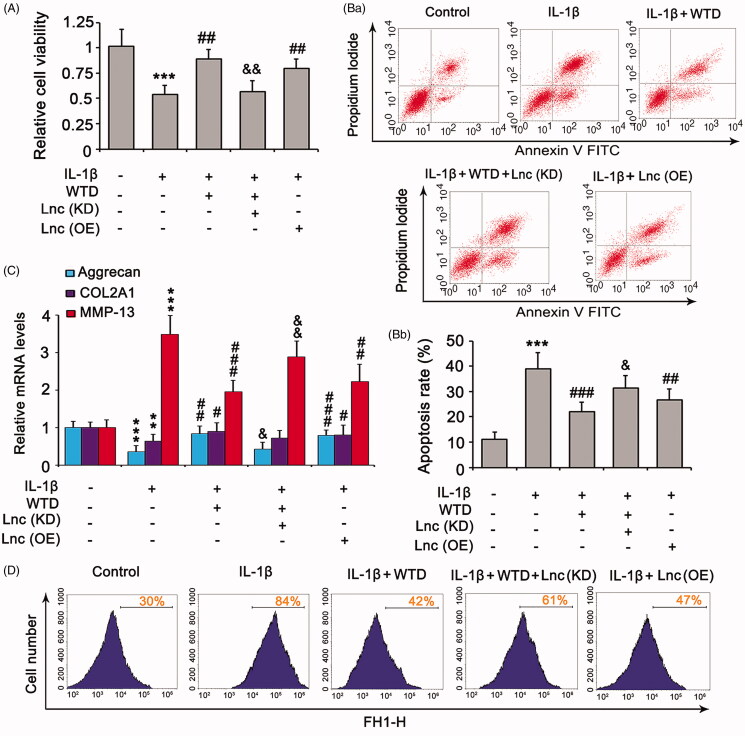
The protective effect of WTD against IL-1β is associated with LOC101928120. CHON-001 cells were treated with 10 ng/mL IL-1β alone or in combination with 1 μg/mL WTD. LOC101928120 was knocked down or overexpressed before the cell treatments. (A) Cell viability was evaluated by the MTT assay. (B) Apoptosis rate was evaluated by flow cytometry. (C) Aggrecan, COL2A1, and MMP-13 expression in chondrocytes was determined by PCR. (D) Intracellular ROS levels were evaluated by a DCFH-DA probe using flow cytometry. ***p < 0.001 vs. control group; ^#^*p* < 0.05, ^##^*p* < 0.01, and ^###^*p* < 0.001 vs. IL-1β group. ^&^*p* < 0.05 and ^&&^*p* < 0.01 vs. IL-1β + WTD group.

Since LOC101928120 mediates the regulatory effect of WTD on SHC1, we hypothesized that LOC101928120 also participates in the protective effect of WTD against IL-1β. Knockdown of LOC101928120 completely abolished or partially attenuated the protective effects of WTD against IL-1β in cell viability (Figure 6(A)), apoptosis (Figure 6(B)), expression of aggrecan, COL2A1, and MMP-13 (Figure 6(C)), and ROS production (Figure 6(D)). In contrast, overexpression of LOC101928120 suppressed the detrimental effects of IL-1β in these various aspects.

**Figure 7.**
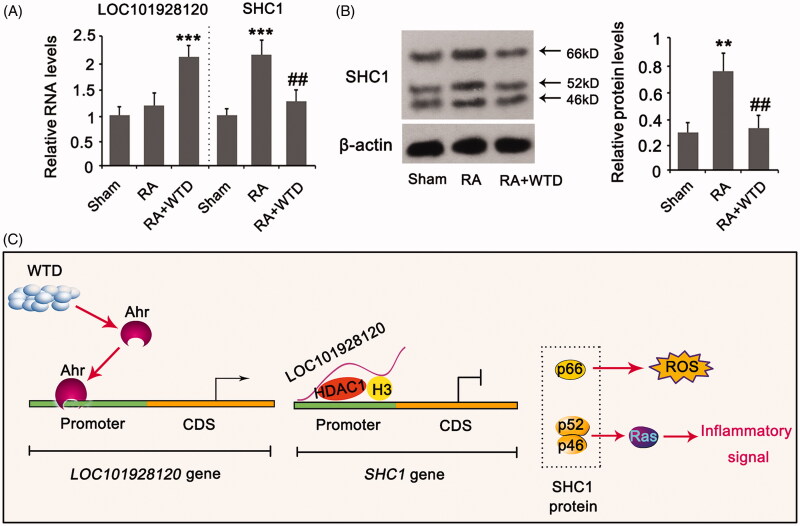
WTD regulated LOC101928120 and SHC1 expression in the articular cartilage of rats in RA. Rats in the RA and RA + WTD groups were injected in the right hind footpad with 0.1 mL complete Freund’s adjuvant containing 10 mg/mL dead *Mycobacterium tuberculosis*. Rats in the RA + WTD group were additionally intragastrically administered WTD crude extract at a dose of 9.8 g/kg/day for 15 days. (A) PCR and (B) western blot assays were performed to determine LOC101928120 and/or SHC1 expression in the articular cartilage of rats. (C) According to the results from this study, we showed the molecular mechanism underlying WTD protection in chondrocytes against IL-1β. **p < 0.001 and ***p < 0.001 vs. control group; ^##^*p* < 0.01 vs. IL-1β group.

Both the mRNA and protein levels of SHC1 were increased in the articular cartilage of rats in the RA group compared to the sham group (*p* < 0.001 and *p* < 0.01, respectively, Figures 7(A,B)). Treatment with WTD increased LOC101928120 in the articular cartilage of rats with RA (*p* < 0.001 vs. sham group) and suppressed the increase of SHC1 mRNA and protein levels (*p* < 0.01 vs. the IL-1β group). Figure 7(C) shows the molecular mechanism underlying WTD protection in chondrocytes against IL-1β. In the mechanism, WTD induces the activation of Ahr, which translocate to the nucleus and induces LOC101928120 transcription. LOC101928120 recruits HDAC1 to the promoter region of the *SHC1* gene and induces the deacetylation of histone 3, resulting in the suppression of the *SHC1* gene. Since p66SHC1 primarily induced ROS production and apoptosis, and p46SHC1 and p52SHC1 induce inflammatory signalling (e.g., Ras signal), suppression of SHC1 attenuated the detrimental effects induced by IL-1β.

## Discussion

WTD increased the expression of LOC101928120 by stimulating Ahr-mediated transcription. LOC101928120 recruited HDAC1 to the histone 3 protein, resulting in the deacetylation of this protein. Deacetylation of histone 3 is associated with the suppression of *SHC1* gene expression. Consequently, the detrimental effects of IL-1β in chondrocytes were attenuated by the suppression of *SHC1* expression. WTD reportedly has multiple biological effects against RA. WTD inhibits angiogenesis in experimental arthritis by blocking VEGFR2 signalling (He et al. 2018). As a result, the expression of IL-1β, IL-17, transforming growth factor-β, platelet-derived growth factor, placental growth factor, angiopoietin (Ang) I, and Ang II were reduced in the synovium of RA rats (He et al. 2018). Zhang et al. (2016) reported that WTD induced thermogenesis by modulating the peroxisome proliferator-activated receptor γ-pathway, which can alleviate RA. The present data reveal a novel mechanism by which WTD attenuates RA by modulating the Ahr/LOC101928120/SHC1 pathway.

The *SHC1* gene encodes three main isoforms: p66Shc, p52Shc, and p46Shc. Isoform p66Shc is a downstream target of the tumour suppressor p53 and is indispensable for the ability of stress-activated p53 to induce elevation of intracellular ROS, cytochrome c release, and apoptosis. Shin et al. (2020) found that depletion of p66Shc suppressed ROS in human chondrocytes in response to monosodium iodoacetate and ameliorated inflammatory cytokine production and cartilage damage in an arthritis model *in vivo*. The isoforms p46Shc and p52Shc, but not p66Shc, are well-known activators of the Ras signalling cascade. Ras signalling may induce inflammation in RA (de Launay et al. 2010). Apart from Ras signalling, p46Shc and p52Shc also induce inflammation through nuclear factor-kappa B (NF-κB) signalling (Lin et al. 2013). In this study, IL-1β induced the increase of ROS as well as apoptosis in chondrocytes. However, treatment with WTD attenuated these detrimental effects of IL-1β. WTD reversed the increase in SHC1 induced by IL-1β. Knockdown of SHC1 also attenuated these detrimental effects of IL-1β in chondrocytes. These results suggest that the protective effects of WTD against IL-1β are probably associated with the suppression of SHC1. Inhibition of Ras signalling failed to restore the viability of chondrocytes that were suppressed by IL-1β but hindered the effect of IL-1β on aggrecan, COL2A1, and MMP-13 expression. It has been confirmed that IL-1β-induced inflammatory signals decrease the levels of cartilage components, such as aggrecan and COL2A1, but increases enzymes (e.g., MMP-13) that degrade cartilage (Yuan et al. 2016; Zhao et al. 2019). These effects collectively induce the destruction of cartilage. Inhibition of the SHC1/Ras pathway may disrupt IL-1β-induced inflammatory signalling and protect cartilage.

Accumulating evidence indicates that lncRNAs are involved in the pathogenesis of RA due to their roles in the regulation of autoimmunity and inflammation-related processes (Zhang et al. 2017; Yang et al. 2018). LncRNA HOTAIR blocks the activation of the NF-κB pathway by sponging miR-138, thereby alleviating inflammation in RA (Zhang et al. 2017). In contrast, the lncRNA NTT/PBOV1 axis induces monocyte differentiation, which promotes inflammation in RA (Yang et al. 2018). LncRNAs regulate gene expression through multiple mechanisms, such as epigenetics, alternative splicing, small RNA sponging, and transcriptional and translational regulation. This study focused on LOC101928120 because the *LOC101928120* gene in the sense strand is near the *SHC1* gene in the antisense strand. The *SHC1* gene is thought to be regulated by LOC101928120, as LOC101928120 can recruit transcriptional regulatory factors to the promoter region of the gene. Indeed, this study in the RNA-pull down assay confirmed that LOC101928120 during the 100 to 400 bp region can bind to the HDAC1. HDAC1 is responsible for the deacetylation of histone 3. Deacetylation of histone 3 is associated with the suppression of gene transcription. For example, Lnc-Smad3 interacts with the histone deacetylase HDAC1 and then silences Smad3 transcription (Xia et al. 2017). LncRNA-SATB2-AS1 recruits p300 and induces acetylation of histone 3, resulting in upregulation of SATB2 (Wang et al. 2019). *SHC1* gene expression is vulnerable to the acetylation status of histone 3. By ChIP assay, we further found that LOC101928120 recruited HDAC1 to the promoter region of the *SHC1* gene and induced the deacetylation of histone 3. A similar effect was also observed with WTD treatment. This effect was abolished after the knockdown of LOC101928120. These data suggest that WTD upregulated LOC101928120 to recruit HDAC1 to *SHC1* gene promoter, thereby suppressing SHC1 expression.

Bioinformatics analysis indicated that LOC101928120 is more likely regulated by Ahr. This speculation was further confirmed by the luciferase reporter assay. Ahr is a ligand-activated transcription factor involved in multiple biological and pathological processes, including immune function, obesity, intestinal homeostasis, and carcinogenesis (Jeuken et al. 2003; Jarry et al. 2005; Tian et al. 2019). Ahr signalling has been implicated in the metabolism of environmental toxicants (Che and Dai 2019). However, many Ahr ligands (e.g., flavonoids, indoles, and resveratrol) are present in natural food and herbal medicines, such as green tea, broccoli, mushrooms, soybean, propolis, and ginseng, suggesting multiple beneficial effects exerted by Ahr in the host (Furue et al. 2014; Lv et al. 2018; Wang et al. 2018; Tian et al. 2019). When binding to the ligands, Ahr moves from the cytoplasm to the nucleus and consequently stimulates the transcription of target genes. Presently, WTD induced the translocation of Ahr from the cytoplasm to the nucleus. As indicated by the ChIP assay, Ahr binds to the promoter region of LOC101928120 and promoted its expression. Therefore, WTD increased the expression of LOC101928120 by stimulating Ahr-mediated transcription.

There are a few limitations to this study. We did not identify that Ahr mediates the protection of WTD. Ahr is a transcriptional factor implicated in various biological functions. Therefore, knockdown of Ahr alone might be harmful to chondrocytes, not to mention the treatment together with IL-1β. In addition, this study did not overexpress SHC1 to determine whether it can abolish the protective effect of WTD against IL-1β. The sequence of the *SHC1* gene is very long. Therefore, it is difficult to overexpress this gene.

## Conclusions

In conclusion, treatment with WTD alleviated RA in rats induced by CFA and reversed IL-1β-induced apoptosis, inflammatory response, and ROS production in chondrocytes. The protection of WTD in chondrocytes was associated with an indirect suppressive effect on *SHC1* expression. WTD increased the expression of LOC101928120 by stimulating Ahr-mediated transcription. LOC101928120 recruited HDAC1 to *SHC1* gene promoter to suppress its expression.
